# Coordinated loss of microRNA group causes defenseless signaling in malignant lymphoma

**DOI:** 10.1038/srep17868

**Published:** 2015-12-07

**Authors:** Makoto Yamagishi, Harutaka Katano, Tsunekazu Hishima, Tatsu Shimoyama, Yasunori Ota, Kazumi Nakano, Takaomi Ishida, Seiji Okada, Toshiki Watanabe

**Affiliations:** 1 Graduate School of Frontier Sciences, Department of Computational Biology and Medical Sciences, The University of Tokyo, Japan; 2 Department of Pathology, National Institute of Infectious Diseases, Japan; 3 Department of Pathology, Tokyo Metropolitan Cancer and Infectious Diseases Center Komagome Hospital, Japan.; 4 Department of Clinical Medical Oncology, Tokyo Metropolitan Cancer and Infectious Diseases Center Komagome Hospital, Japan; 5 Institute of Medical Science, The University of Tokyo, Japan; 6 Center for AIDS Research, Kumamoto University, Japan

## Abstract

Biological robustness is exposed to stochastic perturbations, which should be controlled by intrinsic mechanisms; the promiscuous signaling network without appropriate alleviation is the true nature of cancer cells. B cell receptor (BCR) signaling is a major source of gene expression signature important for B cell. It is still unclear the mechanism by which the expression of functionally important genes is continuously deregulated in malignant lymphomas. Using RISC-capture assay, we reveal that multiple BCR signaling factors are persistently regulated by microRNA (miRNA) in human B cells. Clinical samples from patients with diffuse large B-cell lymphoma (DLBCL, n = 83) show loss of an essential miRNA set (miR-200c, miR-203, miR-31). Conventional screening and RISC profiling identify multiple targets (CD79B, SYK, PKCβII, PLCγ1, IKKβ, NIK, MYD88, PI3K class I (α/β/δ/γ), RasGRP3); signaling network habitually faces interference composed by miRNA group in normal B cells. We demonstrate that simultaneous depletion of the key miRNAs enhances translation of the multiple targets and causes chronic activation of NF-κB, PI3K-Akt, and Ras-Erk cascades, leading to B cell transformation. This study suggests that compensatory actions by multiple miRNAs rather than by a single miRNA ensure robustness of biological processes.

For an effective humoral immune response, mature B cells must recognize foreign antigens and generate antigen-specific effectors. B cell receptor (BCR) signaling is a major source of gene expression signature important for B cell survival, functions, and development^1^. Physiologically, signals from further binary inputs are combined and converted irreducible network with key elements including Erk, Akt, and NF-κB. The integrated signaling amplitude should be equilibrated; when chronically activated by genetic perturbations or other mechanisms, BCR signaling has been accepted as a stem in the pathogenesis of malignant lymphoma/leukemia^2^. DLBCL is the most common aggressive lymphoid neoplasm. Clinical and molecular characteristics, including AIDS, EBV, and expression pattern (e.g. Germinal center B cell like (GC) or non-GC), result in disparate prognoses^3^. DLBCL with more aggressive phenotypes often associates with BCR signaling activation due to upregulation of key signaling factors. However, it is still unclear the mechanism by which the expression of functionally important genes is continuously deregulated.

A biological network is systematic. Robustness and homeostasis of the system are ensured by hierarchical buffering effects against stochastic perturbations^4^. Gene expression is tightly and spatiotemporally regulated by transcription factors, whose activities are provided from the momentary fluctuations and magnitude and spread are effectively amplified by signaling pathways. microRNAs (miRNAs), an emerging class of intrinsic buffering molecules that have diverse functions in mainly post-transcriptional regulation^5^, have been suggested to play pivotal roles in regulation of signaling components^6^. Dynamic and specific alteration of the miRNA pattern observed in cancers strongly suggests the giant roles of this group of molecules. In particular, global downregulation of miRNAs is epigenetically conserved in several neoplasms^7^,^8^.

In mouse B cells, crucial roles of miRNA are genetically demonstrated by *Dicer*-deficient models^9^,^10^. In contrast, the miRNA importance in human B cells is largely uncharted. Deciphering the function of individual miRNAs is still challenging because they are frequently present as families of redundant genes. Their species-specific functions are experimentally and predictively designated^11^. An approach to globally explore miRNA importance in lymphomagenesis makes use of direct comparison of the “functional miRNA” that is validly defined by miRNA-RISC (miRISC) association^12^. Along with quantitative evaluation, a corresponding set of experiments of the physiologically active miRNA group is required to clarify the integrated contribution of miRNA to biological processes of interest.

Several miRNAs have expression patterns that distinguish DLBCL from non-malignant B cells^13^,^14^,^15^. Previous studies have indicated that the altered miRNA expression contributes to lymphoma cell characteristics through activation of signaling pathways including BCR pathway^9^,^10^,^15^,^16^,^17^. However, the molecular interface between miRNA group and signaling factors important for B cell activation has not been characterized. In this study, by reverse engineering of normal and clinical samples, we show an intrinsic defense network composed of a programmed miRNA group. Screening of the RISC-associated functional miRNA and experimental verification demonstrate that important miRNA set is coordinately lost in malignant lymphoma cells. The multi-layered miRNAs appear to be critical for the homeostasis of signaling pathways, including NF-κB, Akt, and ERK pathways. Our findings suggest that the functional compensation by each miRNA ensures robustness of biological systems.

## Results

### miRNAs regulate BCR signaling

**Figure 1:**
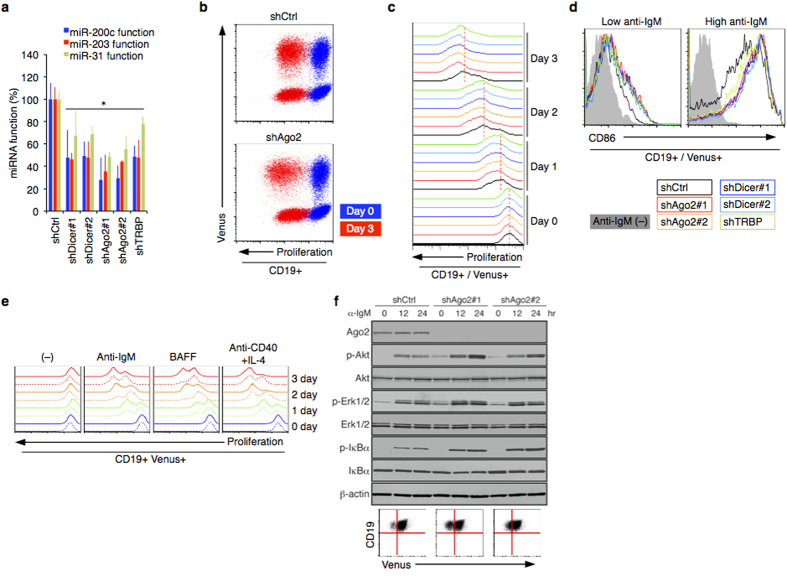
miRNA function in human B cell. (**a**) Inhibition of miRNA biogenesis affects miRNA function. Depletion of Dicer, Ago2, or TRBP was accomplished by specific shRNA. The influence on miRNA function was calculated by luciferase reporter with miR-200c, miR-203, and miR-31 target sequences in 3′UTR (n = 3). Each knockdown decreased endogenous miRNA function and increased Luciferase activity in 293T cells. Up to 80% of knockdown efficiency was confirmed by qRT-PCR (data not shown). (**b,c**) Inhibition of miRNA biogenesis affects proliferation of human primary B cells induced by anti-IgM (5 μg/ml). Dynamic proliferation was examined for 3 days in CD19 + B cells with shRNA targeting RISC components. The shRNA expression was monitored by Venus expression. Representative results (n = 3) of Venus/proliferation (**b**) and time-course (**c**) are shown. Cell proliferation was estimated by CellVue Claret Far Red Fluorescence. Reciprocal relationship between the fluorescence and cell numbers was confirmed (Supplementary Fig. 1a). (**d**) Surface CD86 expression in each shRNA-expressing populations (CD19+/Venus +) under the treatment of anti-IgM (0.1 and 5 μg/ml). (**e**) Inhibition of miRNA biogenesis affects B cell proliferation induced by various stimuli (anti-IgM, 5 μg/ml; BAFF, 0.2 μg/ml; anti-CD40, 2 μg/ml, IL-4, 20 ng/ml). Dynamic proliferation was examined for 3 days in CD19+B cells with shAgo2 (solid lines) and shCtrl (dashed lines). (**f**) Knockdown of Ago2 affects signaling activities in primary B cells assessed by western blotting. Lentivirus transduction was confirmed by Venus expression.

The perspective roles of miRNA in human B cell are unclear. We designed shRNAs targeting Ago2, Dicer, and TRBP^18^, which are responsible for miRNA biogenesis and function. Inhibition of miRNA biogenesis by knockdown (KD) of the RISC factors affected cellular miRNA functions (Fig. 1a). The lentivirus vectors encoded the Venus fluorescent protein, which allows us to monitor the transduced, shRNA-expressing cells. We also stained the cells with a far-red fluorescent dye, which halves in intensity at each cell division, to track cell proliferation (Supplementary Fig. 1a). Depletion of the miRNA machinery accelerated BCR engagement-mediated cell proliferation in human primary B cells (Fig. 1b,c). This experimental evaluation was confirmed in T cell activation with miR-31, which has been identified as a NIK suppressor^8^. Lentivirus-mediated miR-31 or shNIK expression suppressed CD25 expression and cell proliferation induced by T cell receptor stimulation in resting CD4 + T cells (Supplementary Fig. 1b,c). The miRNA inhibition enhanced induction of B cell activation marker CD86 under the condition of BCR engagement (Fig. 1d). In addition, the Ago2 depletion supported B cell proliferation induced by other B cell stimulants such as BAFF and anti-CD40 + IL-4 (Fig. 1e). Western blotting revealed that Ago2 depletion promoted BCR-triggered activation of B cell signaling cascades in human B cells, including Akt, Erk, and NF-κB (Fig. 1f). Thus, the sum total of cellular miRNAs function is suppressive to B cell activation process.

### Screening of functional miRNA by RISC-capture assay

To visualize the interface between miRNA and the BCR pathway, we managed RISC-capture assay, in which RISC immunoprecipitation enabled us to quantitatively survey the functionally and physiologically acting miRNAs and their targeted mRNAs in cells^19^,^20^,^21^,^22^. Because the biological significance of the miRNA-mRNA interaction is still challenged, we performed several control experiments using previously identified miRNA-mRNA interaction and optimized the experimental conditions.

**Figure 2:**
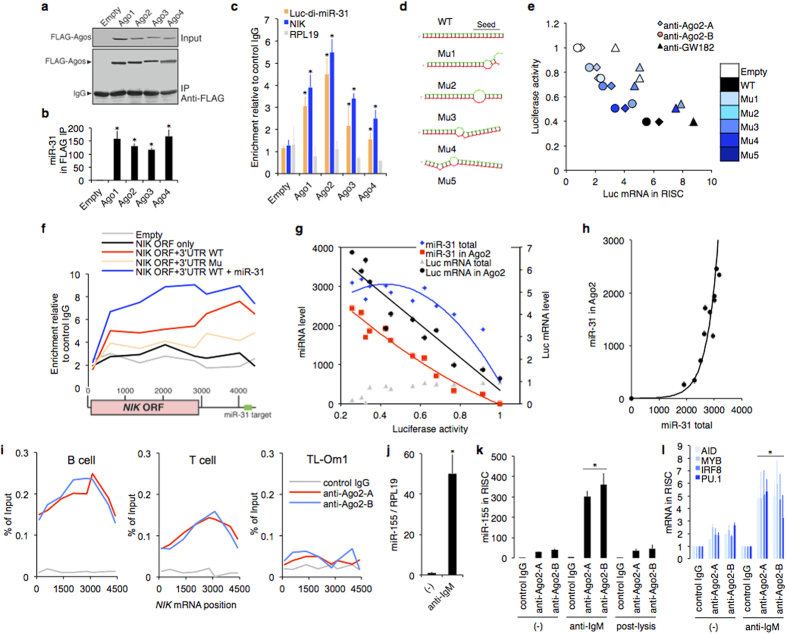
Optimization of RISC-capture assay. (**a–c**) Argonaute family proteins and miR-31 were ectopically expressed in MDA-MB-231 stably expressing *Luc-di-miR-31* (MDA-MB-231/Luc-di-miR-31). Immunoprecipitation of Ago1–4 was performed by anti-FLAG antibody (**a**) and then co-precipitated miR-31 (**b**), *luciferase* mRNA carrying 2 × miR-31 binding sites (*Luc-di-miR-31*), endogenous *NIK* mRNA, and *RPL19* mRNA (as a negative control) (**c**) were quantified by qRT-PCR (n = 3). (**d,e**) WT and five mutated miR-31 (Mu1-5) were introduced in MDA-MB-231/Luc-di-miR-31 cells. Secondary structure analysis by mfold program confirmed stem-loop structure of the pre-miR-31 mutant series expressed by lentiviral vectors. RNAhybrid model represents hybridization between the designed miR-31 series and its corresponding perfect match sequence (**d**). Scatter plot provides relationship between luciferase activity (index of suppression, n = 3) and RISC-incorporated mRNA level (index of mRNA recognition, n = 3) (**e**). Symbol shapes represent antibodies used for immunoprecipitation. Symbol colors represent miR-31 condition. (**f**) NIK expression vector (ORF or ORF + 3′UTR) and/or miR-31 vector were co-transfected in 293T cells. RISC-capture assay with anti-Ago2 antibody was performed and then co-precipitated *NIK* mRNA was quantified by eight distributed PCR primers (n = 3). (**g,h**) MDA-MB-231/Luc-di-miR-31 cells were transfected with various amounts of miR-31-encoding plasmids. Total and RISC-captured levels of miR-31 and *luciferase* mRNA were evaluated by qRT-PCR (n = 3). Luciferase activity (n = 3) means miR-31-dependent suppressive effect. Left scatter plot (**g**) represents the relationship among luciferase activity, miR-31 level, and *luciferase* mRNA level. Right graph (**h**) shows relationship between total and RISC-incorporated miR-31 level. (**i**) RISC-capture assay in normal B cells, T cells, and ATL cell line TL-Om1 (n = 3). Immunoprecipitated *NIK* mRNA level was evaluated by qRT-PCR with the eight distributed primer pairs. (**j–l**) Control experiment of RISC-capture assay in resting and activated B cells (n = 3). miR-155 expression was induced by BCR stimulation in CD19 + B cells (**j**). RISC-incorporated miR-155 was observed under the B cell activation process (**k**). Previously validated miR-155 target mRNAs were captured in RISC of activated B cell (**l**).

First, we co-expressed human Argonaute family proteins and miR-31 in breast cancer cell line MDA-MB-231 stably expressing *Luciferase* mRNA with 2 × miR-31 binding sites in 3′UTR (*Luc-di-miR-31*), which was used for the control experiments because of absence of endogenous miR-31 (reference 23). We performed immunoprecipitation of Ago proteins by anti-FLAG antibody and then quantified co-precipitated miR-31, *Luc-di-miR-31*, and endogenous *NIK* and *RPL19* (as a negative control) mRNAs. We selected Ago2 as bait for RISC capture based on the target abundance (Fig. 2a–c). miRNA specificity in recognition and effectiveness of gene interference were confirmed by designed miRNA mutants. Effective incorporation of target mRNA in RISC led to sufficient suppression of the target expression (Fig. 2d,e). *NIK* mRNA was incorporated into the RISC in the case of WT 3′UTR conjugation, indicating that miRNA recognized a 3′UTR target sequence specifically (Fig. 2f). Correlation among functional miRNA level, Ago2-captured mRNA level, and effectiveness of gene interference was observed. Expression of the captured mRNA was suppressed in an miRNA-dependent manner (Fig. 2g–h).

This method captured endogenous miRNA and its well-known target mRNAs in normal lymphocytes. *NIK* mRNA was detected in Ago2 complex from normal B and T lymphocytes, but not from ATL cell line TL-Om1 that showed NIK overexpression and miR-31 loss^8^ (Fig. 2i). In CD19 + B cells, miR-155 expression was induced by BCR stimulation. The miR-155 and previously validated miR-155 target mRNAs^24^,^25^ were captured in RISC of activated B cells. Post-lysis incubation (between whole lysate from activated B cell and RISC from resting B cell) failed to capture the miR-155 in RISC. Fixation of cells failed to quantify mRNA abundance reproducibly because of mRNA degradation (data not shown). Thus, we concluded that the method could identify and quantify the functional miRNAs and their specific target mRNAs in a biologically relevant context.

**Figure 3:**
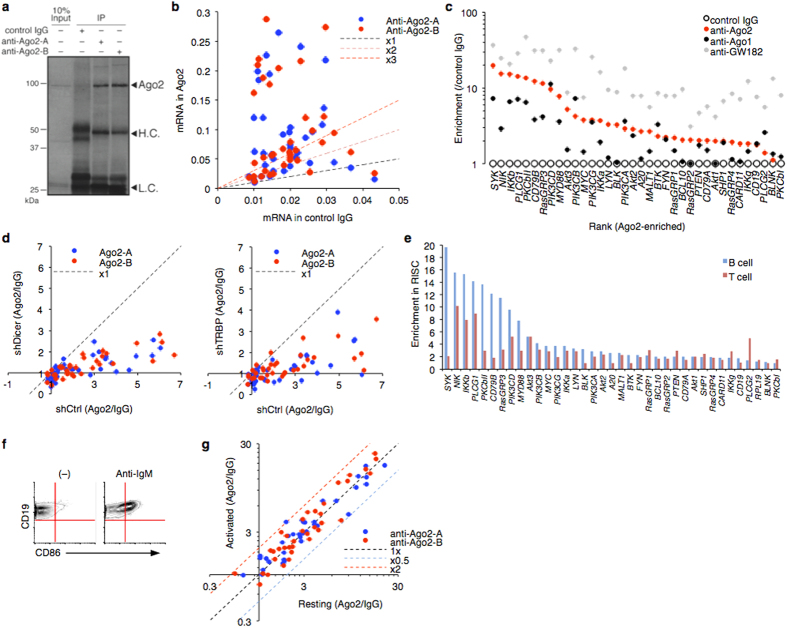
miRNAs-dependent regulation of BCR signaling factors in human B cell. (**a,b**) RISC-capture assay with two independent Ago2 antibodies in human primary B cells. Immunoprecipitated Ago2 was analyzed by western blotting (**a**). H.C. indicates heavy chain of IgG. L.C. indicates light chain of IgG. BCR factor mRNAs (listed in Supplementary Table 1) were quantified by qRT-PCR (**b**). Average values of three independent experiments are shown. Dashed lines indicate reference lines. (**c**) BCR mRNA enrichment in Ago2, Ago1, and GW182 complexes from human B cells assessed by RISC-capture assay. (**d**) RISC-capture assay in Dicer- or TRBP-knockdowned LCL. Scatter plots show RISC-associated mRNA levels in BCR cascade (shDicer or shTRBP vs. shCtrl). Representatives of two independent experiments are shown. (**e**) RISC-capture assay for BCR signaling factors in primary B cells and T cells. Average values of three experiments are shown. The targeted BCR factor mRNAs were less expressed and inefficiently incorporated in RISC in T cells. (**f,g**) B cell activation does not affect miRNA-dependent regulation of BCR factors. CD19 + B cell was activated by anti-IgM for 72 hours. The activation status was confirmed by CD86 expression (**f**). Scatter plot shows RISC-associated levels of mRNA in BCR cascade (resting vs. activated) (**g**). Representative of two independent experiments is shown.

We purified the RISC-RNA complex from human B cells and quantified captured mRNAs for determining the nature of BCR cascades^2^ (Supplementary Table 1). Several mRNA entities were significantly enriched in RISC purified with two independent antibodies (Fig. 3a–c). The candidates were categorized as positive modulators of BCR signaling. The same results were obtained for B cells from other healthy donors (see below). GW182 and Ago1 antibodies showed a similar tendency. Almost all captured mRNAs were released by KD of Dicer and TRBP, supporting that they were constantly recognized by miRISC (Fig. 3d). The BCR factors were less expressed in other peripheral blood lineages, and their mRNAs were inefficiently captured in miRISC (Fig. 3e). The mRNA capture was not due to BCR activation (Fig. 3f,g). Thus, the BCR interference by miRNAs was a homeostatic feature of B cells that confers potent buffer effects, given that Ago2 KD accelerated B cell activation (Fig. 1).

### miRISC is reprogrammed in lymphoma cells

**Figure 4:**
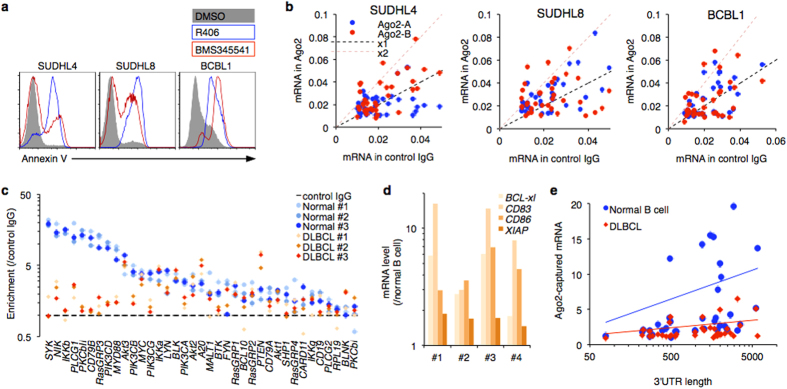
Defective miRNA functions in DLBCL. (**a**) Cell survival of lymphoma cell lines depends on BCR signaling. SUDHL4, SUDHL8, and BCBL1 cells were treated with SYK inhibitor R406 (1 μM) or IKKβ inhibitor BMS345541 (5 μM) for 3 days. The apoptotic cells were detected by Annexin V staining. (**b**) RISC-capture assays in B cell lymphoma cell lines. Two independent anti-Ago2 antibodies were used for RISC immunoprecipitation. Immunoprecipitated levels of BCR factor mRNAs are plotted. Representatives of two independent experiments are shown. (**c**) Quantification of RISC-associated mRNA in normal resting B cells (n = 3) and DLBCL samples (n = 3) assessed by RISC-capture assay. mRNA inputs were comparable among all tested samples (data not shown). (**d**) BCR target gene upregulation in frozen DLBCL samples used in *ex vivo* experiments (RISC-capture assay and Polysome analysis). Expression levels of BCR signaling-inducible genes relative to normal resting B cells are shown. (**e**) Scatter plot showing relationship between 3′UTR length and RISC incorporation efficiency in BCR factor mRNAs. Average values of the RISC-incorporated mRNA levels from DLBCL samples (n = 3) and normal B cells (n = 3) are plotted.

To determine the biological significance of miRNA-mediated interference, we investigated spontaneously BCR-activated models. We tested two BCR signaling inhibitors (SYK inhibitor R406 and IKKβ inhibitor BMS345541) in three B cell lymphoma cell lines derived from GCB-DLBCL (SUDHL4, CD10 +/BCL6 +), ABC-DLBCL (SUDHL8, CD10−/BCL6−) and primary effusion lymphoma (BCBL1, CD10−/BCL6−). The drug treatments significantly induced apoptotic death, indicating that BCR signaling is important for the survival ability of these cell models (Fig. 4a). We performed RISC-capture assay in the lymphoma cell lines and found fewer BCR factor mRNAs in their RISC (Fig. 4b). The RISC-capture assay also demonstrated that several kinds of mRNA showed less inclusion in RISC from BCR-activated DLBCL biopsy samples (n = 3; DLBCL#1 is GC-DLBCL; DLBCL#2 and DLBCL#3 are non-GC-DLBCL) than in normal B cells (Fig. 4c,d). Previously identified PTEN and A20 regulation by miRNA was extracted in DLBCL samples^26^,^27^, suggesting that an intact RNA-protein complex was conserved in the frozen samples. Longer 3′UTRs tended to be recognized by miRISC (Fig. 4e).

**Figure 5:**
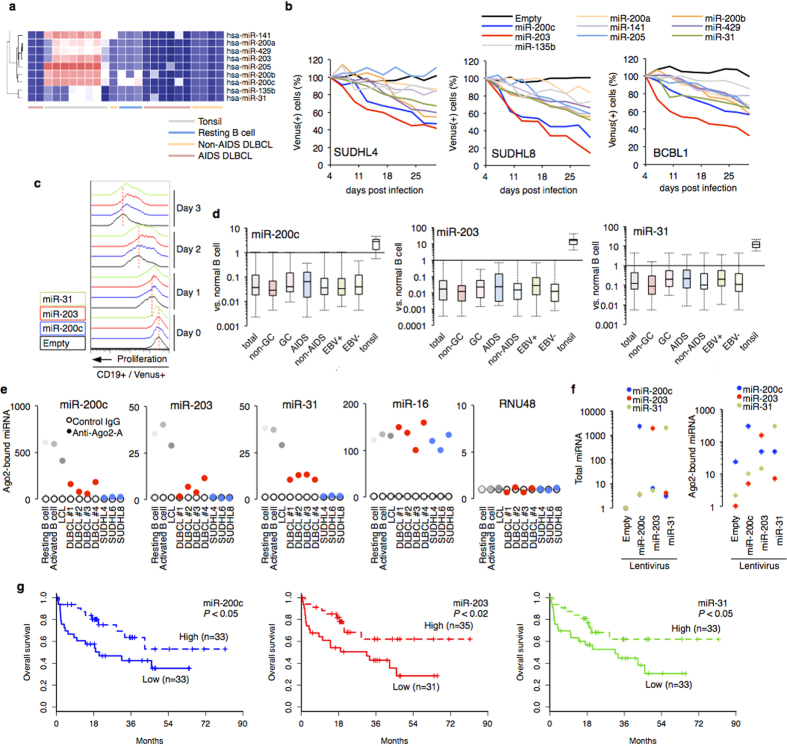
Functional loss of miR-200c, miR-203, and miR-31 in DLBCL cells. (**a**) Hierarchical clustering of miRNA expression in AIDS-associated DLBCL (n = 8), non-AIDS DLBCL (n = 5), tonsil (n = 8), and normal CD19 + B cell (n = 3). (**b**) Lymphoma cell lines were transduced with lentivirus vectors carrying pri-miRNA (or pre-miRNA, data not shown), followed by long-term culture in complete medium. Results as percentage of each Venus-positive population transduced with shRNA series are plotted (n = 3). (**c**) miRNA expression affects proliferation of human CD19 + B cells induced by anti-IgM (5 μg/ml). miRNA expression was monitored by Venus expression. Representative results of proliferation time-course experiments (n = 3) are shown. (**d**) miRNA quantification in DLBCL cohort (total, n = 83; GC, n = 31;, non-GC, n = 34; AIDS, n = 20; non-AIDS, n = 63, EBV + , n = 30; EBV−, n = 48) and tonsil (n = 10). CD19 + B cells (n = 3) were used for normal control samples. (**e**) RISC-associated miRNA levels in normal B cells (n = 3, mean values), LCL (n = 3, mean values), primary DLBCL samples (n = 4), and three DLBCL cell lines evaluated by RISC-capture assay. (**f**) Transduction of miRNA lentivirus could express functional miRNA in lymphoma cells. Graphs show the levels of total miRNA (left) and RISC-incorporated miRNA (right) in SUDHL8 with miRNA lentivirus (n = 3, average values). (**g**) Overall survival probabilities of DLBCL cases with high miRNA (dashed line) and low miRNA (solid line).

We paid close attention to miRNAs that showed decreased levels in DLBCL, because mRNAs of key BCR factors showed less inclusion in lymphoma RISC. We performed miRNA microarray analysis of primary DLBCL samples. Unbiased clustering identified an miRNA group that showed significant decrease in lymphoma cells compared with normal resting B cells and tonsils (Fig. 5a). The focal cluster (low expression in lymphoma) included miR-200 family (miR-200a, miR-200b, miR-200c, miR-141, and miR-429), miR-203, miR-205, miR-135b, and miR-31. A similar result has been reported in another B cell lymphoma cohort^28^. We expressed the candidate miRNAs by lentivirus vectors in the BCR-addicted lymphoma cells and analyzed each effect on the lymphoma cell proliferation. In particular, miR-200c, miR-203, and miR-31 commonly showed significant effects (Fig. 5b). To further examine the role of the miRNAs, we introduced each miRNA in human resting B cells and then analyzed BCR-mediated cell proliferation. The results showed that miR-200c, miR-203, and miR-31 prevented B cell activation (Fig. 5c).

Quantitative RT-PCR (qRT-PCR) analysis of a large cohort (n = 83) confirmed that these miRNAs were expressed in B cells and drastically suppressed in lymphoma with various clinical features (Fig. 5d). The RISC-capture assay showed obvious loss of functional miRNAs in lymphoma cell lines and biopsy samples compared with normal B cells (Fig. 5e). miRNA abundance directly affected potential miRISC formation (Figs 2h and 5f). These results demonstrated that BCR signaling-regulatory miRNAs are coordinately lost in DLBCL. The loss of miRNAs was associated with poor prognosis in DLBCL (Fig. 5g).

### miRNA has multi-interface in BCR signaling

**Figure 6:**
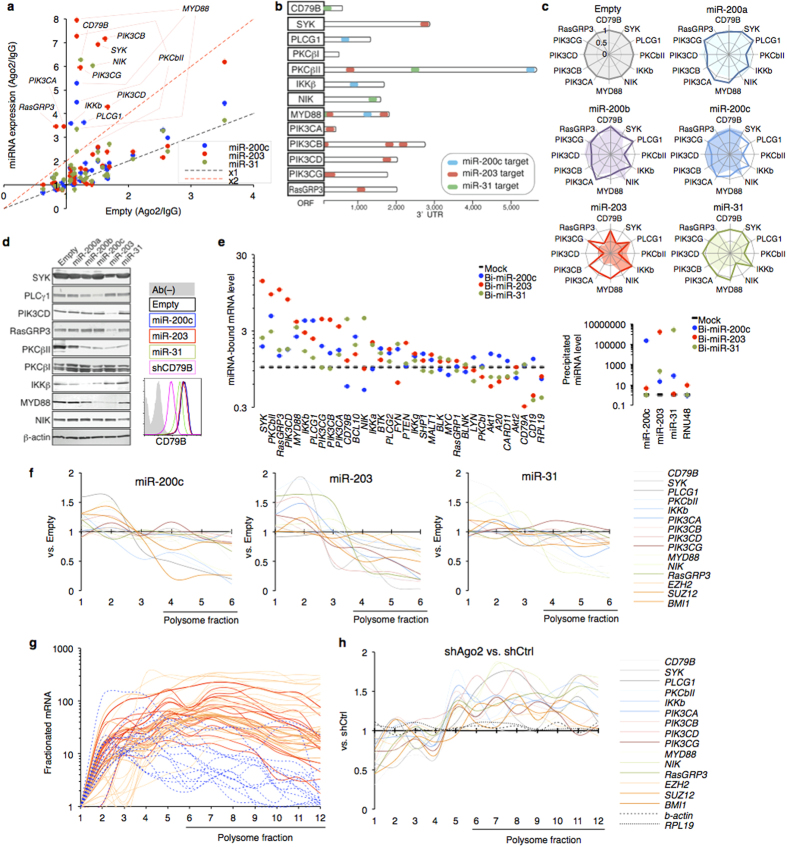
Overlapped regulatory network by miRNA. (**a**) Scatter plot showing RISC-associated mRNA levels of BCR signaling factors (SUDHL8/miRNA vs. SUDHL8/Empty). Average values of three independent experiments are shown. (**b**) Schematic of the miRNA target sites in 3′UTR of the BCR signaling factors based on predictions by computational algorithms and results of RISC-capture screening, 3′UTR reporter assay, qRT-PCR, and western blotting. (**c**) Radar charts showing association between miRNA and targets in BJAB cells. Solid lines indicate mRNA level affected by miRNA expression (n = 3); filled lines indicate 3′UTR luciferase value (n = 3~4). (**d**) BCR factors expression in miRNA-expressing SUDHL8 analyzed by western blotting and flow cytometry (for CD79B). NIK was visualized by MG132 pretreatment. (**e**) Pull-down assay with biotinylated miRNA (Bi-miRNAs). Bi-miRNAs or mock were transfected into BJAB cells. The biotinylated miRNA and targeted mRNA complex were pull-downed by streptavidin beads. The incorporated miRNA (right graph) and mRNA of BCR factors (left graph) were quantified by qRT-PCR (n = 3, mean values). (**f**) mRNA quantification of sucrose-gradient fractions in miRNA-expressing SUDHL8. The fractionated mRNA levels (miRNA expression vs. Empty) are plotted. Representatives of two independent experiments are shown. (**g**) Polysome analysis of DLBCL samples (n = 3, solid lines) and B cell (dashed line). The mRNA levels of BCR factors in each fraction were quantified by qRT-PCR and calculated as relative to the lightest fraction (no.1). Line colors indicate different individuals. (**h**) Polysome analysis in shAgo2 or shCtrl-expressing LCL. BCR mRNA levels in each fraction were quantified. Representative of two independent experiments is shown.

For screening of target genes of the variable miRNAs (miR^3^: miR-200c, miR-203, and miR-31), we expressed each miRNA in SUDHL8 cells and then quantified mRNA incorporated in the miRNA-abundant RISC (Fig. 6a). This survey and computational algorithms provided each miRNA-mRNA interaction (Fig. 6b and Supplementary Table 2). We observed the biological miRNA-mRNA interaction in B cells by conventional 3′UTR reporter assay, mRNA quantification (Fig. 6c), and western blotting (Fig. 6d). Direct associations between the miRNA and target mRNA were demonstrated by pull-down assay with biotinylated miRNA (Fig. 6e). In summary, miR-200c targeted PLCγ1, PKCβII, IKKβ, and MYD88; miR-203 targeted SYK, PKCβII, MYD88, PI3K class I (α/β/δ/γ), and RasGRP3; and miR-31 targeted CD79B, PKCβII, MYD88, and NIK in B cells. Their mRNAs were less enriched in lymphoma RISC than in normal RISC (Fig. 4). Of note, other cell types showed ineffective enrichment of the mRNA in RISC despite high miR^3^ level (Fig. 3e and data not shown).

qRT-PCR revealed that the miRNA could not degrade the almost transcripts (Fig. 6c), prompting us to examine the effect of miRNA on translational regulation. Translational profiling is reproducibly accomplished by polysome analysis on a sucrose gradient (Supplementary Fig. 2a–f). We found that miR^3^ inhibited translation initiation of the verified target genes (Fig. 6f). Reciprocally, the targeted BCR mRNAs were massively associated with polysome from lymphoma biopsy, directly indicating the active translation of BCR factors in DLBCL (Fig. 6g).

The gradient fractions from B cells revealed that RISC factors and miR^3^ were associated with translation machinery (Supplementary Fig. 2c,d). Ectopically expressed Argonaute family proteins were detected in (poly-)ribosome fractions (Supplementary Fig. 2g). Immunoprecipitated RISC and ribosomal proteins were bound to miR^3^ in B cells, suggesting that miR^3^ participates in translational regulation (Supplementary Fig. 2h). We performed Ago2 KD and then analyzed mRNA in the fractions. Ago2 deficiency led to active translation of BCR factors (Fig. 6h). Collectively, miRNA was found to be involved in translational regulation of BCR factors.

### miRNAs regulates BCR signaling pathways

**Figure 7:**
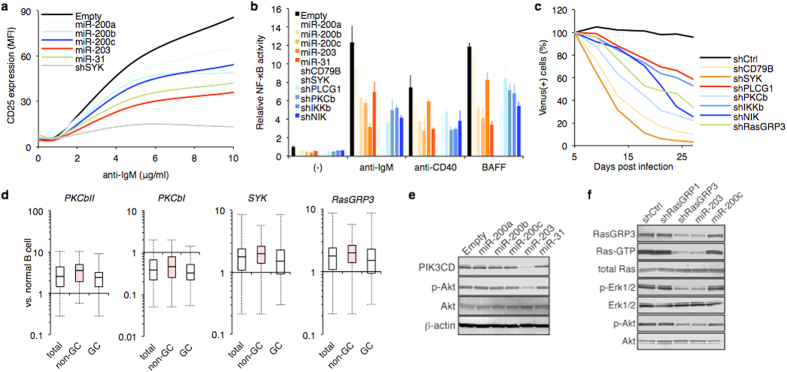
miR^3^ function in BCR signaling regulation. (**a**) BJAB cells were transduced with miRNA or shSYK-encoding lentivirus, followed by stimulation with anti-IgM at indicated concentrations for 2 days. CD25 expression level (mean fluorescence intensity, MFI) was analyzed by flow cytometry. (**b**) miR^3^ suppresses NF-κB activation in B cell. BJAB cells with miRNA or shRNA targeting BCR factor were treated with indicated B cell stimulants for 24 hours. Relative NF-κB activity was analyzed by 6 × κB luciferase assay (n = 3). (**c**) Depletion of BCR signaling factors suppresses B cell lymphoma cell proliferation. SUDHL8 cells were treated with shRNA-expressing lentivirus, followed by long-term culture in complete medium. Results as percentage of each Venus-positive population transduced with shRNA series are plotted. Similar result was observed in SUDHL4 cells (data not shown). We confirmed that the knockdown efficiencies were comparable among the shRNAs (mean fold change vs. shCtrl is 0.255 ± 0.09 in 293T cells). (**d**) Results of qRT-PCR in DLBCL cohort (total, n = 68; GC, n = 31; non-GC, n = 35) shown as value relative to normal B cells. (**e**) Western blots showing total and phosphorylated Akt and PIK3CD (p110δ) levels in SUDHL8/miRNA cells. (**f**) Western blots showing cellular levels of RasGRP3, Ras-GTP (active form of Ras), total Ras, phosphorylated and total Erk1/2 and Akt in SUDHL8/shRasGRP1, /shRasGRP3, /miR-200c, and /miR-203 cells.

The emerging interaction between miRNAs and target genes suggested a buffering role of miR^3^ on the B cell signaling pathways. Indeed, miR^3^ prevented BCR-triggered B cell activation (Fig. 5c and Fig. 7a). Individual depletion of BCR factors by specific shRNAs or miRNA expression inhibited receptor-triggered NF-κB activity and induced lymphoma cell death in which BCR signal was activated, suggesting that upregulation of the signaling elements caused chronic activation (Fig. 5b and Fig. 7b,c). BCR proximal components such as SYK and CD79B showed stronger effects in this process. miR-203 suppressed *SYK* at the mRNA and protein levels (Fig. 6). Our cohort and a previous study^29^ showed *SYK* mRNA upregulation in DLBCL (Fig. 7d).

PKCβ has been implicated in multiple B cell functions^30^. *PRKCB* encodes two isoforms, PKCβI and PKCβII, whose mRNA have differential 3′UTR (Fig. 6b). miR^3^ could target only PKCβII through the long 3′UTR. A high *PKCβII* level was validated in the DLBCL cohort (Fig. 7d).

The PI3K-Akt pathway is crucial in B cell proliferation and survival^31^. *PIK3CD* (p110δ) had nonredundant roles in Akt activation and lymphoma survival^32^,^33^. miR-203 regulated all members of the p110 family (Fig. 6) and thus inhibited p-Akt level in lymphoma cells (Fig. 7e).

RasGRP3, a member of Ras guanyl nucleotide exchange factors, directly affects Ras activity and downstream phosphorylation of Erk and Akt in B cells^34^. miR-203 recognized and downregulated *RasGRP3* but not other *RasGRP* at the mRNA and protein levels (Fig. 6). miR-203 expression led to low Ras activity and resulted in low phosphorylation of Erk1/2 in lymphoma cells (Fig. 7f). qRT-PCR revealed *RasGRP3* overexpression in DLBCL cohort (Fig. 7d).

### Coordinated loss of miRNAs enhances B cell transformation

**Figure 8:**
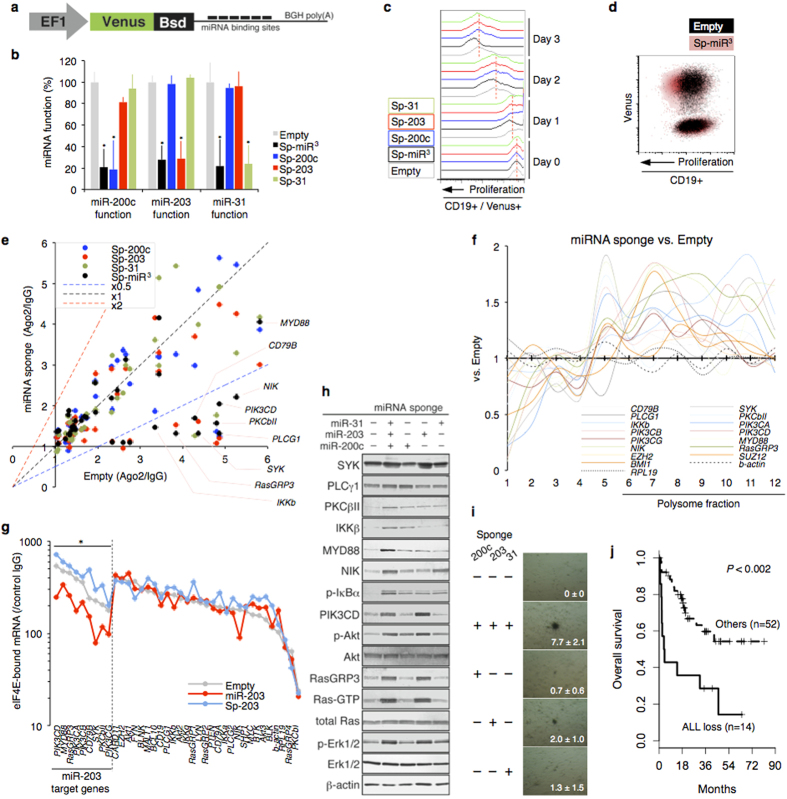
miRNA-mediated cooperative interference and its capacity. (**a**) Schematic of miRNA-sponge construct. 7 × miRNA binding sites were inserted into *Venus* 3′UTR. (**b**) miRNA sponge-mediated inhibition of endogenous miRNA. The miRNA function was evaluated by activity of luciferase with miR-200c, -203, -31 target sequences in 3′UTR (n = 3). (**c,d**) Proliferation time-course (c) and a representative result of Venus/proliferation on day 3 (d) in CD19 + B cells with miRNA sponge under the anti-IgM treatment are shown (n = 3). (**e**) Scatter plot of RISC-associated mRNA levels in BCR signaling (LCL/miRNA-sponge vs. LCL/Empty). Average values of three independent experiments are shown. (**f**) Polysome analysis showing translation efficiency of BCR signaling factors in LCL with miRNA-sponge or empty. Representative of two independent experiments is shown. (**g**) miRNA affects eIF4F complex on mRNAs of BCR signaling. Graph shows results of RNA-immunoprecipitation assay with anti-eIF4E antibody in miR-203-modulated LCLs (n = 3, mean values). Co-precipitated mRNAs of BCR signaling factors were analyzed by qRT-PCR and shown as relative values (vs. control IgG). (**h**) Western blots showing BCR factors and phosphorylation cascade in miRNA-sponge treated LCL. (**i**) A high-sensitivity agar cloning assay was performed by embedding LCL with miRNA sponge. Representative results and the mean ± SD (n = 3) of colony numbers are provided. (**j**) Overall survival probabilities of DLBCL cases with low expression of miR3 (ALL loss, solid line) and other cases (Others, dashed line) (log-rank test P < 0.002).

The miRNA-orchestrated overlapping interactome suggested multiple regulatory windows in signaling pathways, which implied their compensatory roles. We established miRNA inhibitory sponge lentivirus, enabling us to specifically and stably inhibit the endogenous miRNA set (Fig. 8a,b). We found that simultaneous depletion of miR^3^ enhanced B cell activation (Fig. 8c,d). Importantly, sole inhibition failed to activate B cells sufficiently. Loss of the cooperatively acting miRNAs had additive effects on the release of some common targets from RISC (Fig. 8e). Similar to the result of Ago2 KD, miR^3^ deficiency caused active translation of the BCR factors (Fig. 8f). RNA-immunopurification with an anti-eIF4E antibody revealed that BCR factor translation was cap-dependent. The eIF4F-mRNA complex was disassembled by miRNA (Fig. 8g).

Western blots demonstrated the orchestrated expression of the targeted genes and synchronous acquisition of NF-κB, PI3K-Akt, and Ras-Erk activities by sustained miR^3^ loss (Fig. 8h). Altogether, miR^3^ shapes a network of cooperative gene interference (CGI). The redundant functions of miRNAs may be required for the complementary actions; in concordance, miR^3^ and regulated BCR mRNA were lost in lymphoma RISC. Defective miRNA-mediated CGI conferred colony formation ability (transformation) in LCL, an untransformed B cell that self-propagates by EBV infection (Fig. 8i). The coordinated loss of the key miRNAs was significantly associated with poor clinical outcome in DLBCL (Fig. 8j, log-rank test P < 0.002)

These results collectively demonstrated that miRNA-mediated buffering function is critical for B cell activation and cellular phenotypes. Coordinated inactivation of miRNAs enhances BCR signaling and B cell transformation.

## Discussion

Through the functional screening, the molecular interface between miRNA and signaling factors important for B cell activation has been elucidated, which supports the CGI concept. Although some miRNAs are involved in the activation of BCR signaling^35^, the perspective direction of miRNA is clearly suggested to be a negative modulator of activating signals, a result consistent with previous observations^8^,^10^,^36^. The important challenge is why miRNA acts as a buffer. The answer may be provided by a cancer cell. The defenseless signaling is the true nature of the cancer cell. Our most striking finding is that malignant cell potentially avoids the buffering effect.

The RISC-capture assay has demonstrated that several components within the interconnected signaling pathways are under constant surveillance by multi-layered miRNAs. Stable, orchestrated alteration of functional miRNA levels contributes to chronic activation of signaling pathways that is frequently observed in cancer cells. According to the mounting evidence, individual miRNAs have small effects on multiple key targets; in reality, the compensatory actions by multiple miRNAs rather than by a single miRNA ensure robustness against stochastic perturbations. Taken together, the signaling cascade habitually confronts interference composed by miRNA group. All tested lymphoma samples showed loss of the essential miRNA set, suggesting that the defenseless signaling is a prerequisite for lymphoma development.

By in-depth investigation of human primary samples and clinical samples such as by RISC-capture assay, polysome analysis, and qRT-PCR, we obtained unparalleled results. Interestingly, miRNAs, particularly those affecting B cell signaling, were modestly expressed in B cells, which is the possible reason why they have not yet been investigated. However, the RISC-loaded functional levels were significantly changed depending on B cell status. It is highly recommended that functionally acting miRNA should be emphasized for precise understanding of miRNA significance.

We report the unprecedented molecular traits of B cell lymphoma: the functionally dead miRNA set, key targetable signaling factors, and their regulatory mechanisms. Primary tumors directly showed overexpression of the key miRNA targets; they include essential BCR proximal factors (SYK, CD79B) and key signaling modulators (PKCβII, IKKβ, PI3K family (p110), PLCγ1, NIK, MYD88, RasGRP3), all of which are suggested as crucial factors for physiological and pathological BCR signaling^1^,^2^,^31^,^32^,^33^,^34^,^37^,^38^,^39^,^40^,^41^,^42^,^43^,^44^, and upregulation of these factors and underlying mechanism have been mostly unidentified until now. We note a direct relationship between BCR factor abundance and conclusive signaling magnitude. Quantitative analysis of the set of miRNAs may be a practical tool to evaluate BCR signaling activation and prognosis in DLBCL (Figs 5g and 8j).

We have perceived that miRNA rather affects translational activity. Polysome analysis combined with miRNA manipulation has clearly indicated translation initiation as the miRNA-responsive step. RNA immunoprecipitation assay with eIF4E antibody has indicated that miRNA disrupts the complex between 5′-capped mRNA and eIF4F. Recent studies have shown that eIF4A is a main target of miRNA-dependent translation blockade^45^,^46^. In addition to the transcriptome, increasing translation activity appears to be a hallmark of the B cell lymphoma. A gene expression signature definitively including the proteome is again important for the classification and molecular targeting of malignant cells. The upregulation of BCR factors may support chronically sustained signaling, which produces cumulative signals^47^.

The key miRNAs in B cells including miR-200c, miR-203, and miR-31 are recognized as tumor-suppressive miRNAs in other cancers^8^,^48^,^49^,^50^,^51^,^52^,^53^. Interestingly, common epigenetic mechanisms are suggested in the cancer-associated downregulation of the miRNAs. Further elucidating the regulatory mechanisms of the essential miRNAs may be of critical importance for the understanding of molecular pathogenesis of malignant lymphomas, which are closely associated with global epigenetic alterations^54^,^55^.

## Methods

### Cell culture

Primary peripheral blood mononuclear cells (PBMC) were isolated by Ficoll separation (Ficoll-Paque, GE Healthcare). Normal CD19 + B cells and CD4 + T cells were prepared from human PBMC by B Cell Isolation Kit II and CD4 + T Cell Isolation Kit, respectively (Miltenyi Biotec). The immunopurified cells were confirmed by flow cytometry. DLBCL cell lines SUDHL4, SUDHL6, and SUDHL8 were purchased from DSMZ. Adult T cell leukemia (ATL) patient-derived TL-Om1 cell line was a gift from Dr. K. Sugamura, Tohoku University, Japan. B95.8 EBV-transformed lymphoblastoid cell lines (LCLs) were previously established by infection of lymphocytes from four independent healthy donors with culture supernatants of the virus producer B95.8 line^56^. All lymphoid cell lines and primary lymphocytes were cultured in RPMI1640 (GIBCO) supplemented with 10% of FBS (GIBCO) and antibiotics (GIBCO). 293T, 293FT, and MDA-MB-231 cells were maintained in DMEM (Nissui, Japan) with 10% of FBS and antibiotics.

B cell activation was performed by anti-IgM (Jackson Immunoresearch Laboratories), BAFF (R&D Systems), anti-CD40 (R&D Systems), and/or IL-4 (R&D Systems). T cell activation was performed by anti-CD3/CD28 antibodies (Miltenyi Biotec).

### Clinical samples

Frozen biopsy and formalin-fixed paraffin-embedded (FFPE) tissues from patients with DLBCL or acute tonsillitis were used in this study. The DLBCL tissues were chosen randomly from a collection of specimens obtained during the course of diagnostic procedures. The DLBCL cases were diagnosed according to the WHO classification^3^ and subgrouped into the germinal center (GC) B-cell or non-GC B cell molecular type based on the Hans immunohistochemistry algorithm^57^. Other clinical information (EBV+/− and AIDS+/−) was also considered to link the *ex vivo* data and the clinical status. Informed consent was obtained from patients after 2007. Overall survival was determined from the date of diagnosis to the date of death of any cause. Survival curves were computed by the Kaplan-Meier method and compared by the log-rank test. The study and use of human subjects were approved by research ethics committees of the University of Tokyo, National Institute of Infectious Disease, and Tokyo Metropolitan Cancer and Infectious Diseases Center Komagome Hospital. The methods were carried out in accordance with the approved guidelines of the journal.

For quantification of the specific mRNA, DNaseI-treated total RNA from frozen tissue and primary lymphocytes were used. For quantification of the miRNA, DNaseI-treated total RNA from frozen tissue, FFPE, and primary lymphocytes were used. Total RNA from FFPE samples were prepared using PureLink FFPE RNA Isolation Kit (Invitrogen) and the RNA quality was checked by Bioanalyzer system (Agilent Technologies). Frozen biopsy specimens were also directly used in the RISC-capture assay, Polysome analysis, and quantitative RT-PCR.

### Quantitative RT-PCR

Total RNA isolation was performed by ISOGEN (Wako, Japan). DNaseI-treated total RNA was subjected to reverse-transcriptase (RT) reaction using SuperScript II (Invitrogen) with manufacturer’s protocol. Random primers-based synthesized cDNA was analyzed by quantitative PCR (Thermal cycler Dice, TAKARA). The specific PCR was performed using gene-specific primers (Supplementary Table 3) and SYBRGreen (Applied Biosystems). The levels of *RPL19* or *β-actin* mRNA were also quantified for internal control. Quantification of mature miRNA was performed by MicroRNA Assays (Applied Biosystems). *RNU48* was tested as a control small RNA.

### RISC-capture assay

RISC-capture assay was previously described^19^,^20^,^21^,^22^. The purified B cells, T cells, LCL, cell lines (1 × 10^7^), or the frozen specimens (approximately 1-3 mm^3^) were washed once with PBC and then lysed on ice for 20 min in buffer containing 25 mM Tris-HCl, pH 7.4, 150 mM KCl, 5 mM EDTA, 0.5% Nonidet P-40, 5 mM DTT, protease inhibitors, and 100 units/ml RNase inhibitor. Equal volume of cell lysate were incubated at 4 °C for 2 hours with 5 μg of anti-Ago2 antibodies (A, clone 4G8 mouse monoclonal antibody, Wako, Japan; B, clone 2A8 mouse monoclonal antibody, MBL, Japan), anti-GW182 antibody (Anti-TNRC6A/GW182 rabbit polyclonal antibody, MBL), anti-Ago1 antibody (Anti-EIF2C1/AGO1 rabbit polyclonal antibody, MBL) or mouse control IgG (I5381, SIGMA) antibodies, followed by incubation with 30 μl of protein G sepharose beads at 4 °C for 1 hour. Subsequently, the immunocomplex was washed three times with the ice-cold lysis buffer and then subjected in total RNA isolation. The incorporated mRNA and miRNA were quantified by qRT-PCR with specific primer sets (Supplementary Table 3). The immunoprecipitated protein was analyzed by western blotting with anti-Ago2 antibody (RN005M, MBL).

### Biotinylated miRNA pull-down assay

Biotinylated miRNA (Bi-miRNA) pull-down assay was performed as described^58^ with some modifications. Custom biotinylated miRNAs (Bi-miR-200c, -203, -31) were purchased from Thermo Fisher Scientific. Five nmol of the Bi-miRNAs or mock were transfected into 1 × 10^7^ of BJAB cells by Lipofectamine2000 (Invitrogen). After 18 hours incubation, the transfectants were lysed on ice with RNA-IP buffer (same with RISC-capture assay). The biotinylated miRNA and targeted mRNA complex were pull-downed by streptavidin beads (Dynabeads M-280 Streptavidin, Invitrogen) according to the manufacturer’s instructions. The complexes were washed four times with the ice-cold lysis buffer and then subjected in total RNA isolation. The incorporated mRNA and miRNA were quantified by qRT-PCR.

### Flow cytometry

Single-cell suspensions of cell culture or lymphocytes were stained with fluorescent-labeled antibodies. Anti-CD4 (555349, clone RPA-T4) and CD19 (555413, clone HIB19) antibodies were purchased from BD Biosciences. Anti-CD25 (302609, clone BC96), CD86 (305405, clone IT2.2) and CD79B (341405, clone CB3-1) antibodies were purchased from BioLegend. Apoptosis cells were determined by PE Annexin V/7-AAD staining (BD Biosciences). Cell proliferation was dynamically examined by CellVue Claret staining (CellVue Claret Far Red Fluorescent Cell Linker Kit, SIGMA) with manufacturer’s protocol. The fluorescence and lentivirus-mediated Venus expression were analyzed by flow cytometry using a FACSCalibur (BD Biosciences). The acquired data were analyzed by Flowjo software (Tree Star).

### Western blotting

Western blots were performed with first antibodies listed below; p-Akt (#4060, Cell Signaling), Akt (#9272, Cell Signaling), p-Erk1/2 (#4370, Cell Signaling), Erk1/2 (#4695, Cell Signaling), p-IκBα (#9246, Cell Signaling), IκBα (sc-371, Santa Cruz), SYK (sc-1240, Santa Cruz), PLCγ1 (#5690, Cell Signaling), PIK3CD (sc-7176, Santa Cruz), RasGRP3 (#3334, Cell Signaling), PKCβI (sc-209, Santa Cruz), PKCβII (sc-210, Santa Cruz), IKKβ (sc-8014, Santa Cruz), MYD88 (#4283, Cell Signaling), NIK (#4994, Cell Signaling, protein was visualized by MG132 pretreatment.), FLAG (F3165, SIGMA), β-actin (sc-69879, Santa Cruz), p-RPS6 (#4858, Cell Signaling), RPS6 (#2317, Cell Signaling), RPL19 (sc-1000830, Santa Cruz), Ago2 (RN005M, MBL), GW182 (RN033P, MBL). Ras activation was assayed by comparing the amount of Ras-GTP and total Ras in identical cell lysates. The Ras-GTP was collected by pull-down with GST-Raf (Ras binding domain, Jena Bioscience). Alkaline phosphatase-conjugated anti-mouse and anti-rabbit secondary antibodies were from Promega. The blots were detected by BCIP/NBT substrate (Promega).

### Lentivirus construction and production

Replication-defective, self-inactivating lentivirus vectors were used^8^,^59^. Mature miRNA sequence or shRNA (Supplementary Table 3) were cloned into a CS-RfA-EVBsd vector via pENTR4-H1. For miRNA sponge construction, 6 × tandem sequences of miRNA target were cloned into 3′UTR of Blastcidin resistant gene of CS-RfA-EVBsd vector. The established viral vectors were co-transfected with the packaging plasmid (pCAG-HIVgp) and the VSV-G- and Rev-expressing plasmid (pCMV-VSV-G-RSV-Rev) into 293FT cells. High-titer viral solutions for were prepared by centrifugation-based concentration and used for transduction into cell lines or primary cultures. The infection was attained by spinoculation method (1,800 rpm, 2 hours) and then cultured in appropriate condition for 5 to 7 days. The transduced population was also selected with 10 μg/ml of Blastcidin for 3 days. Only previously validated shRNA sequences (from original articles, siRNA databases, or company websites) were employed in this study. The knockdown efficiency was confirmed by western blotting and/or qRT-PCR.

### miRNA target gene predictions

To identify putative miRNA target genes, we integrated the output results of multiple prediction programs; TargetScan (http://www.targetscan.org/), PicTar (http://pictar.org/), and miRanda (http://www.microrna.org/microrna/). RNAhybrid (http://bibiserv2.cebitec.uni-bielefeld.de/rnahybrid/) was used to obtain the secondary structure of miRNA-3′UTR base pairing.

### 3′UTR-conjugated reporter assay

3′UTR sequences carrying putative miRNA target sites were amplified and inserted into pMIR-REPORT (Ambion) using specific primer sets (Supplementary Table 3). Human B cell line BJAB cells were transduced with miRNA lentivirus and then co-transfected with the 3′UTR-inserted firefly plasmid and RSV-Renilla luciferase plasmid. The cells were collected at 24 h post-transfection, and Dual-luciferase reporter assay was performed (Promega).

### Polysome analysis

The purified B cells, LCL, cell lines (1 × 10^7^), or the frozen specimens (approximately 1–3 mm^3^) were washed with ice-cold PBS, lysed on ice in lysis buffer (10 mM Tris-HCl, pH 7.5, 140 mM NaCl, 1.5 mM MgCl_2_, 0.5% NP-40, 2 mM DTT, 0.5% (w/v) deoxycolate, 100 μg/ml cycloheximide, 100 units/ml RNase inhibitor, protease inhibitor cocktail) for 10 min and then centrifuged at 12,000 × *g* for 10 sec to pellet the nuclei. Polysome fractionations of the cell extract were performed on 11  ml of gradients from 15%–45% (w/v) sucrose in 10 mM Tris-HCl, pH 7.5, 140 mM NaCl, 5 mM MgCl_2_, 1 mM DTT, 100 μg/ml cycloheximide. The lysates were separated by centrifugation at 38,000 rpm for 2 hours in a SW41 rotor (Beckman Coulter) at 4 °C. Each fractionated sample (500 μl each) was measured absorbance at 254 nm for determination of ribosome-associated fractions. For RNA extraction, the each fraction was treated with 40 μg/ml of Proteinase K and 10 mM of EDTA, followed by total RNA isolation by ISOGEN-LS (Wako, Japan). The isolated ribosomal RNA was analyzed by denaturing agarose gel electrophoresis. The mRNA distribution was analyzed by qRT-PCR and calculated as relative to the lightest fraction (no.1). For protein extraction, 100% (w/v) trichloroacetic acid (TCA, Wako) was used. The TCA-precipitated protein samples were analyzed by western blotting.

### RNA immunoprecipitation assay

Purified CD19 + B cells or LCL (1 × 10^7^) were washed once with PBC and then lysed on ice for 20 min in buffer containing 25 mM Tris-HCl, pH 7.4, 150 mM KCl, 5 mM EDTA, 0.5% Nonidet P-40, 5 mM DTT, protease inhibitors, and 100 units/ml RNase inhibitor (TAKARA). Equal volume of cell lysate were incubated at 4 °C for 2 h with 5 μg of anti-eIF4E (RN006M, MBL), anti-RPS6 (#2317, Cell Signaling), anti-RPL19 (sc-100830, Santa Cruz) or control IgG (I5381, SIGMA) antibodies, followed by incubation with 30 μl of protein G sepharose beads at 4 °C for 1 hours. Subsequently, the immunocomplexes were washed three times with the ice-cold lysis buffer and then subjected in total RNA isolation. The incorporated mRNA and miRNA were quantified by qRT-PCR.

### Microarray analyses

For the labeling of miRNAs in the total RNA samples, miRNA Complete Labeling and Hybridization Kit (Agilent Technologies) was used. Labeled miRNA samples were then subjected to hybridization procedure to Human miRNA microarray kit v2 (Agilent Technologies), which has probes for 723 human miRNAs and 73 human viral miRNAs on board. After hybridization, the microarrays were washed and proceeded to detection of the signals by the microarray scanner (Agilent Technologies G2565BA). Scanned spot intensities were converted to values by Feature Extraction software (Agilent Technologies), and analyzed by GeneSpring GX (Agilent Technologies).

### Luciferase assay

Cellular NF-κB activity was evaluated by NF-κB reporter assay as previously described^8^,^60^. Briefly, 6 × κB-luciferase plasmid and control RSV-Renilla plasmid were co-transfected into target cells by Lipofectamine2000 (Invitrogen). At 24 hours post-transfection, the cells were collected and analyzed by Dual-luciferase assay system (Promega). For NF-κB activation, the cells were seeded in 12 well culture plate at the cell density of 5 × 10^5^ cells/1ml/well and treated with indicated stimulants (anti-IgM, 5 μg/ml; BAFF, 0.2 μg/ml; anti-CD40, 2 μg/ml).

### LCL colony formation assay

A high-sensitivity agar cloning assay was performed in triplicate by embedding 2 × 10^3^ LCL cells in 500 μl of RPMI1640 containing 10% FBS and 0.3% soft agar (Difco) on the basal layer containing 0.6% agar in RPMI1640 with 10% FBS in 12 well plate. Medium change was performed every 4–6 days. Colonies were stained with 0.001% of crystal violet (SIGMA) and scored at day 20. Representative results and the mean ± SD of colony numbers are provided.

### Statistical analyses

The statistical significance was tested by two-tailed pared Student’s t-test with *P* < 0.05 (*) for all *in vitro* experiments with cell lines, primary lymphocytes, and clinical samples. Data are represented as mean ± SD unless otherwise noted.

## Additional Information

**How to cite this article**: Yamagishi, M. *et al.* Coordinated loss of microRNA group causes defenseless signaling in malignant lymphoma. *Sci. Rep.* **5**, 17868; doi: 10.1038/srep17868 (2015).

## Supplementary Material

Supplementary Information
