# Supplementary material for: Coordinated loss of microRNA group causes defenseless signaling in malignant lymphoma
Source: Sci Rep. 2015 Dec 7;5:17868. doi: 10.1038/srep17868 (PMC4671098; doi:10.1038/srep17868)
Supplement: Supplementary Information [file srep17868-s1.pdf]

Supplementary Information for

Coordinated loss of microRNA group causes defenseless signaling in malignant lymphoma

Makoto Yamagishi[1*], Harutaka Katano[2], Tsunekazu Hishima[3], Tatsu Shimoyama[4], Yasunori Ota[5], Kazumi Nakano[1], Takaomi Ishida[5], Seiji Okada[6], and Toshiki Watanabe[1*]

[*]Corresponding authors:
Dr. Makoto Yamagishi, Laboratory of Tumor Cell Biology, Department of Medical Genome Sciences, Graduate School of Frontier Sciences, The University of Tokyo, 4-6-1 Shirokanedai, Minato-ku, Tokyo, 108-8639, Japan; E-mail: myamagishi@mgs.k.u-tokyo.ac.jp
Dr. Toshiki Watanabe, Laboratory of Tumor Cell Biology, Department of Medical Genome Sciences, Graduate School of Frontier Sciences, The University of Tokyo, 4-6-1 Shirokanedai, Minato-ku, Tokyo, 108-8639, Japan; E-mail: tnabe@ims.u-tokyo.ac.jp

Affiliations
[1]Graduate School of Frontier Sciences, Department of Computational Biology and Medical Sciences, The University of Tokyo, Japan
[2]Department of Pathology, National Institute of Infectious Diseases, Japan
[3]Department of Pathology and [4]Department of Chemotherapy, Tokyo Metropolitan Cancer and Infectious Diseases Center Komagome Hospital, Japan
[5]Institute of Medical Science, The University of Tokyo, Japan
[6]Center for AIDS Research, Kumamoto University, Japan

Supplementary Figure 1

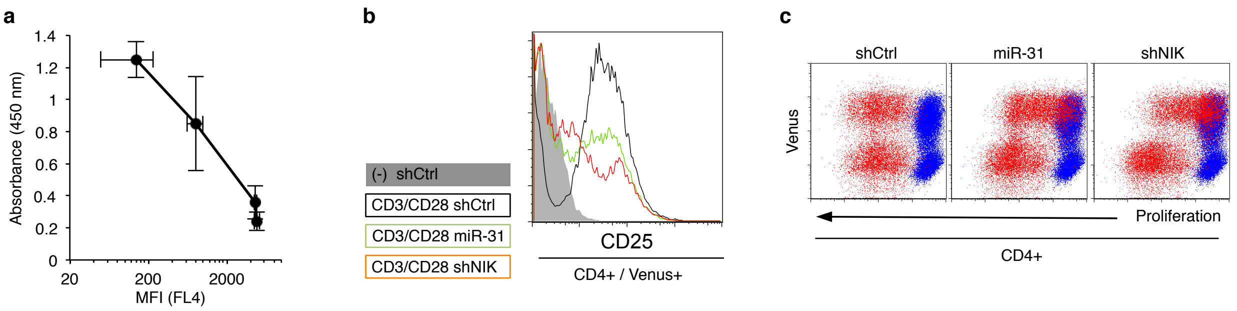

Supplementary Figure 1. miRNA function in human B cell.
(a) Cell proliferation was estimated by CellVue Claret Far Red Fluorescence. Reciprocal relationship between the fluorescence of the CellVue Claret (FL4) and cell numbers (absorbance at 450 nm) was confirmed (n = 3, mean ± SD). (b,c) miR-31 suppresses T cell activation. Lentivirus-mediated miR-31 or shNIK expression suppressed CD25 expression (b) and cell proliferation (c; day 0, blue dots; day 3, red dots) in resting CD4+ T cell in the presence or absence of anti-CD3/CD28 antibodies. Venus expression was used as a marker of lentivirus infection.

Supplementary Figure 2

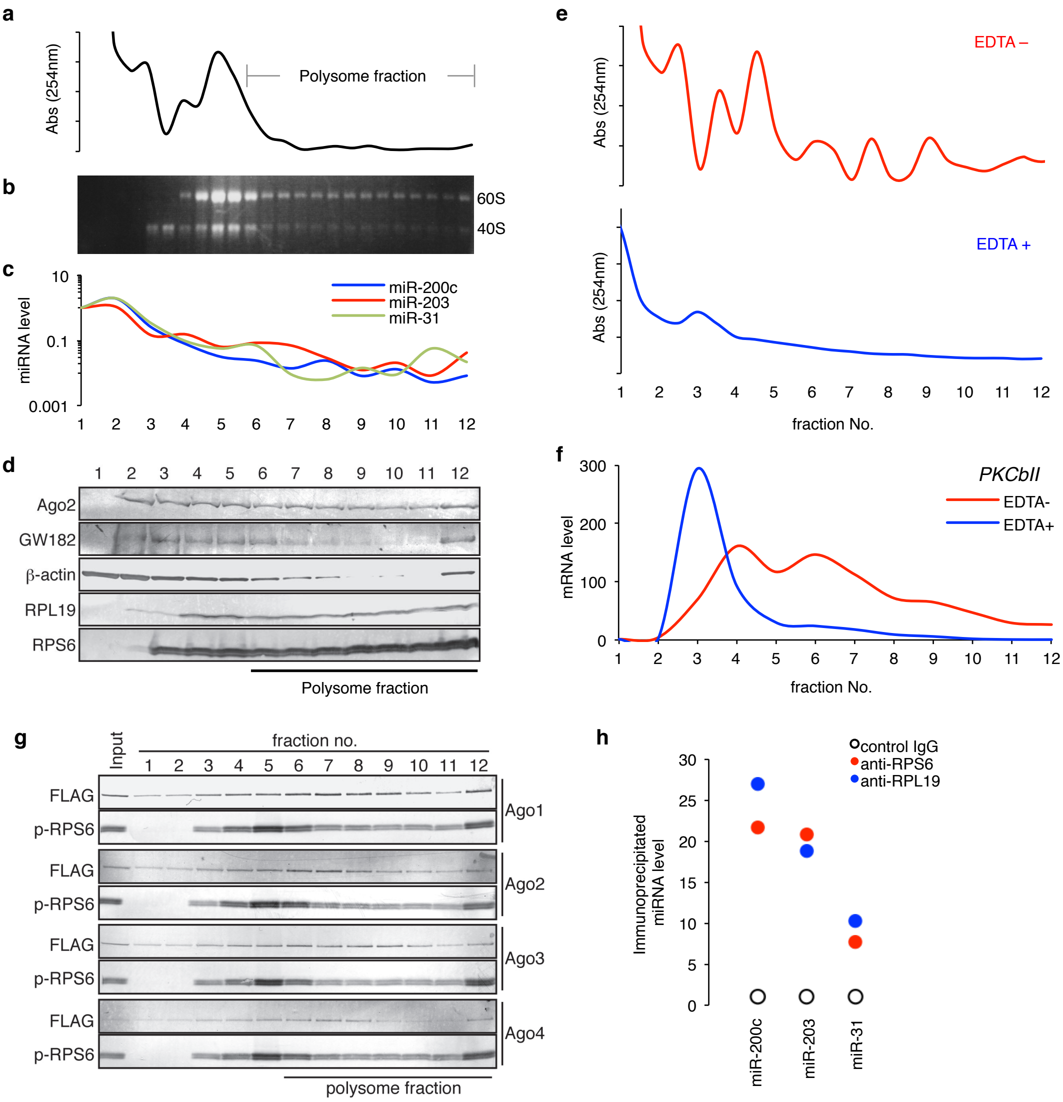

Supplementary Figure 2. miRNA in translational regulation.

(a-d) Translational profiling is reproducibly accomplished by polysome analysis on a sucrose gradient. Cytoplasmic extract from CD19+ B cells was analyzed by ultracentrifugation on 15-45% sucrose density gradients. The fractionated samples were then measured absorbance at 254 nm (a), detected ribosomal RNA by denaturing agarose gel electrophoresis (b), quantified fractionated miRNA by qRT-PCR (c), and analyzed polysome-associated proteins by western blotting with indicated antibodies (d). Freezing the samples at −80°C did not influence the polysome composition in the experimental setting (data not shown). (e,f) Polysome analysis of SUDHL8 cells in the presence (25 mM) or absence of EDTA. Absorbance at 254 nm (e) and and *PKCβII* mRNA level (relative to fraction no.1) (f) of each fraction are shown. (g) Human Argonaute family proteins are associated with ribosome. 293T cells were transfected with FLAG-tagged Ago1-4 expression plasmids. At 48 hours post transfection, the cells were analyzed by 15-45% sucrose density gradient sedimentation. The total proteins from the fractionated samples were examined by western blotting with anti-FLAG and anti-p-RPS6 antibodies. (h) miRNA is associated with ribosomal proteins. RNA-immunoprecipitation assay with anti-RPS6 and anti-RPL19 antibodies were performed in CD19+ B cells. The co-precipitated miRNA were evaluated by qRT-PCR.

Supplementary Table 1. **Interface between miRNA and BCR factor mRNAs.**
The gene list of BCR factors tested in this study (reference 2). Results of RISC-capture assay with anti-Ago2-A, Ago2-B, Ago1, and GW182 antibodies in normal B cell (n = 3, average value relative to control IgG) are provided.

No.	Gene symble	NCBI No.	3'UTR length	RISC-capture assay in B cell (/control IgG)				Recognizing miRNA
				Ago2-A	Ago2-B	Ago1	GW182	
1	CD19	930	222	1.426	1.615	1.829	7.682	
2	CD79A	973	392	1.466	2.056	1.510	5.724	
3	CD79B	974	488	9.656	15.047	3.864	31.341	miR-31
4	BLK	640	491	3.302	3.782	1.041	8.934	
5	LYN	4067	1721	3.838	3.755	1.210	9.435	
6	FYN	2534	1271	2.421	3.019	2.104	7.965	
7	SYK	6850	2960	16.944	21.581	7.311	36.969	miR-203
8	BLNK	29760	274	1.709	1.101	1.345	13.436	
9	BTK	695	438	1.361	2.938	3.335	8.563	
10	PLCG1	5335	1208	16.301	19.453	7.141	28.603	miR-200c
11	PLCG2	5336	296	0.949	1.664	2.602	5.578	
12	PKCbI	5579	498	0.362	0.588	1.250	8.026	
13	PKCbII	5579	5795	15.844	12.218	6.498	47.330	miR-200c,-203,-31
14	CARD11	84433	562	1.668	1.660	1.072	7.201	
15	BCL10	8915	1850	3.756	2.656	1.070	9.613	
16	MALT1	10892	2296	3.436	3.959	1.472	5.642	
17	IKKa	1147	1231	4.745	4.128	1.952	16.314	
18	IKKb	3551	1674	12.011	16.188	6.589	20.938	miR-200c
19	IKKg	8517	585	1.598	2.144	1.456	12.393	
20	PIK3CA	5290	345	3.758	2.698	3.666	18.095	miR-203
21	PIK3CB	5291	2702	3.580	4.993	7.346	33.044	miR-203
22	PIK3CD	5293	2068	10.565	9.487	11.392	23.072	miR-203
23	PIK3CG	5294	1760	4.662	4.185	3.551	25.772	miR-203
24	PTEN	5728	3303	2.754	2.181	1.744	4.667	miR-214
25	SHP1	825047	272	1.893	2.427	1.901	6.685	
26	Akt1	207	991	1.949	1.647	1.006	4.183	
27	Akt2	208	3555	3.696	3.771	2.349	7.934	
28	Akt3	10000	5530	5.097	3.711	1.545	8.725	
29	MYD88	4615	1869	7.336	9.222	3.591	32.008	miR-200c,-203,-31
30	MYC	4609	476	6.175	4.208	1.432	7.532	
31	NIK	9020	1515	18.059	19.077	2.920	24.900	miR-31
32	RasGRP1	10125	2453	2.347	2.138	1.493	7.901	
33	RasGRP2	10235	227	5.179	2.861	1.001	3.106	
34	RasGRP3	25780	1994	13.606	12.403	4.173	31.152	miR-203
35	RasGRP4	115727	982	4.840	3.121	1.150	8.454	
36	A20	7128	1993	3.017	2.263	1.157	7.915	miR-125b

Supplementary Table 2. Details of miRNA targeted mRNA.

This table provides the results of RISC-capture assay with enforced miRNA expression, pull-down assay with biotinylated miRNA, and RNAhybrid modeling between predicted target sequences and miRNA.

Gene Name	RISC-capture assay (vs Empty)			Bi-miRNA Pull-down assay (vs Mock)			RNA Hybrid model	ΔG (kcal/mol)	Recognizing miRNA
	miR-200c	miR-203	miR-31	miR-200c	miR-203	miR-31			
CD79B	1.222	1.383	5.094	0.570	1.241	4.293	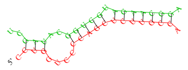	–26.9	miR-31
SYK	1.704	4.608	1.596	1.892	13.584	2.445	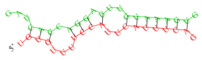	–16.9	miR-203
PLCG1	2.835	0.884	0.934	4.084	2.375	1.338	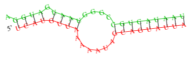	–18.0	miR-200c
PKCβII	3.840	6.234	2.073	4.745	9.063	2.968	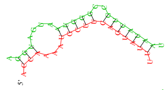	–16.4	miR-200c
							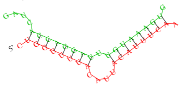	–21.2	miR-203
							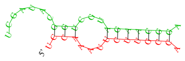	–19.3	miR-31
IKKβ	3.342	1.579	1.132	4.074	2.850	1.634	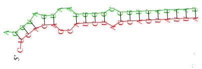	–21.9	miR-200c
NIK	0.788	1.610	4.241	0.510	0.966	4.438	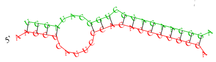	–27.4	miR-31
MYD88	4.552	6.825	2.886	2.540	3.978	3.325	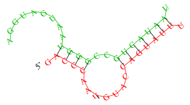	–16.0	miR-200c
							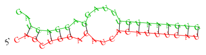	–17.5	miR-203
							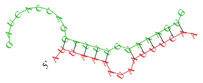	–11.7	miR-203
							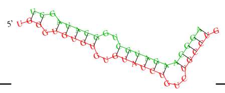	–30.0	miR-31
PIK3CA	0.954	3.590	1.259	1.673	3.464	1.467	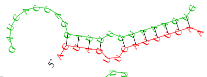	–14.3	miR-203
PIK3CB	1.104	4.417	0.860	1.529	4.209	0.959	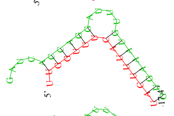	–17.4	miR-203
							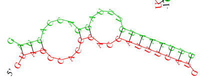	–14.4	miR-203
							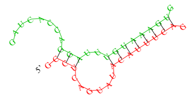	–15.2	miR-203
PIK3CD	0.759	2.583	0.821	1.717	7.819	1.732	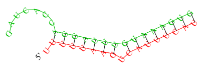	–15.5	miR-203
PIK3CG	0.961	4.863	1.066	1.903	4.292	0.986	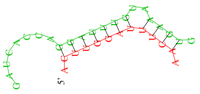	–12.1	miR-203
							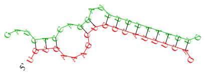	–16.8	miR-203
RasGRP3	0.926	4.143	1.747	1.411	10.406	1.693	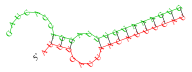	–14.4	miR-203

Supplementary Table 3. PCR primers and oligo DNA used in the study.

Quantitative PCR Primer List (Target Gene: Sequence)

Gene symbol	Forward	Reverse
Ago2	5′-GCC AGC ATA CTA CGC TCA CCT-3′	5′-GTG GTC TCG CCC GTT ACT CT-3′
Dicer	5′-TGC AAA TGT ACC CCG TTC C-3′	5′-ACC TTC CCG TCG TAA GTT CTC TC-3′
TRBP	5′-GGC CTT TCA CGT CAG CTA CC-3′	5′-TGC AGA GCC ATG ACA CAC AG-3′
CD19	5′-TAA TCC CGA TGG GCC AGA-3′	5′-GAA TCT TGG GGA CTT GAG GAG A-3′
CD79A	5′-GGA AAC GAT GGC AGA ACG A-3′	5′-ATG GAG CAG TCG TCC AGG TT-3′
CD79B	5′-CAT CGT GCC TAT CTT CCT GCT-3′	5′-CCA CTT CAC TTC CCC TGT CC-3′
BLK	5′-GGG TCT TCA CCA TCA AAG CAG A-3′	5′-TCC AGG TTG CGG ATG ACC T-3′
LYN	5′-ACG GCT CCA GAA GCA ATC A-3′	5′-GGT CAT CAC GTC GGC ATT AG-3′
FYN	5′-CAA AGG AAG AGT GCC ATA CCC-3′	5′-GCG TTC TTC AGG GTC CTT TT-3′
SYK	5′-GCA GGG TGT CCA AGA GAG ATG-3′	5′-TGC GGG AGC GGT TAG TT-3′
BLNK	5′-CCC AGA AAC AAA TCC ACC AAA-3′	5′-CAT ACC ATG GCT TGC AGA GAA C-3′
BTK	5′-CAT GTA CAG TTG CTG GCA TGA G-3′	5′-GGG GCT TGT GGA GAA GAG AA-3′
PLCG1	5′-AGG AGC ATC TCG CAG ACC A-3′	5′-ATT CTA CAA GGC GCA CAG CA-3′
PLCG2	5′-AGC TGT ACC AGG AGA AAT GCA AC-3′	5′-CAC ACA CAC AAT ACC CTT ACA CAC A-3′
PKCbI	5′-GGA GAA ACT TGA ACG CAA AGA GA-3′	5′-CTT TGT CGA AGT TGG AGG TGT C-3′
PKCbII	5′-GGA GAA ACT TGA ACG CAA AGA GA-3′	5′-TCG GGA GGT GTT AGG ACT GG-3′
CARD11	5′-AGT CCA ACA TCT ACC CCA TCG T-3′	5′-GCC TCC AGC TCC TTC TCC TT-3′
BCL10	5′-GGT CTG GAC ACC CTT GTT GA-3′	5′-CTG GAA AAG GTT CAC AAC TGC TAC-3′
MALT1	5′-TCC CAA GCA TTG CCT CTA TAC C-3′	5′-AGG TTT CCC AAC ATT CAC TTC CT-3′
IKKa	5′-ATG AGG CAA ATG AGG AAC AGG-3′	5′-GGG GAC AGT GAA CAA GTG ACA A-3′
IKKb	5′-GGT GGC TGA AGC ACA TAA CCT-3′	5′-GCT CTT CTT CTT CCG TCT GTA ACC-3′
IKKg	5′-CAG GAT CGA GGA CAT GAG GAA-3′	5′-GGC ACT TGG GAC AGC AGA A-3′
PIK3CA	5′-AGT GAT TAG TAA AGG AGC CCA AGA A-3′	5′-GCA TTC CAG AGC CAA GCA-3′
PIK3CB	5′-TCA CTC TCT TTG CGC TGA TGT T-3′	5′-GCT TCT TCT TCA CTC TTC CCT AAT G-3′
PIK3CD	5′-GGG CTT CTC TTC CTC CAC CT-3′	5′-TGC CTC CTC CTC TGT TTT CC-3′
PIK3CG	5′-AAG ACA AGC CCA CAC TTC CA-3′	5′-CTG GGG CAT TCC TGT CAT C-3′
PTEN	5′-AGC GTG CAG ATA ATG ACA AGG A-3′	5′-GAT TTG ACG GCT CCT CTA CTG TTT-3′
SHP1	5′-GAA GAA TGC CCA TGC CAA G-3′	5′-AGG TCA GGA GAC AGC ACA GG-3′
Akt1	5′-CCA TCA CAC CAC CTG ACC AA-3′	5′-TAT CGT CCA GCG CAG TCC A-3′
Akt2	5′-CCA GTC CAT CAC AAT CAC ACC-3′	5′-TGC TGG CCG AGT AGG AGA A-3′
Akt3	5′-TGT CGA GAG AGC GGG TGT T-3′	5′-GCC ATC TTT GTC CAG CAT TAG A-3′
MYD88	5′-CGG ATG GTG GTG GTT GTC T-3′	5′-GGA ACT CTT TCT TCA TTG CCT TGT-3′
MYC	5′-CCT CGG ATT CTC TGC TCT CCT-3′	5′-CGA TTT CTT CCT CAT CTT CTT GTT C-3′
NIK	5′-CGG GTC AAA GTG GGA GAC A-3′	5′-CTG GCA CCT CCA TGT CGT AG-3′
RasGRP1	5′-TCA CCT CAG ACT GCC TAC CTA CC-3′	5′-CAA GCT GGA GGG ATT CTA TTT TCT T-3′
RasGRP2	5′-GGT GGA GGA TGG GGT GTT T-3′	5′-CCT GCT CTG GTT GAA GTA TTT TCT C-3′
RasGRP3	5′-GCA CGG AGT TTG AAC TTG ACC-3′	5′-TTC TCG TCT TGC CCC TTC C-3′
RasGRP4	5′-CCC AGA CTG AAT CCC CAC A-3′	5′-AGT GAG GAA GAG AGG AGA CCA AGA-3′
A20	5′-AGG ATA CTG CCA GAA GTG TTT CAT T-3′	5′-CTT GAG GTG CTT TGT GTG GTT C-3′
b-actin	5′-TGG CAC CCA GCA CAA TGA A-3′	5′-CTA AGT CAT AGT CCG CCT AGA AGC A-3′
RPL19	5′-ACC AAG GAA GCA CGC AAG C-3′	5′-CAG ACA AAG TGG GAG GTT TTA TTT C-3′
NIK R1	5′-CGC AAC ACT CGC TTG GTT-3′	5′-CGT CTT CTC CTT GGC TTT GG-3′
NIK R2	5′-ATG CTA CAG AGG GCA AAA TGG-3′	5′-TGA ACT GCG GGG TGT TTC T-3′
NIK R3	5′-ACA ACG AGG GTG TCC TGC T-3′	5′-AGG GAC AAT TCT GGG TGA GGT-3′
NIK R4	5′-CGC TCT GCC TCA AGA TTG C-3′	5′-TTG ATT TGG CGG TGG ATG T-3′
NIK R5	5′-CGG GTC AAA GTG GGA GAC A-3′	5′-CTG GCA CCT CCA TGT CGT AG-3′
NIK R6	5′-CAC GAT GCT GCC CTG AAA-3′	5′-ATC CTG TTT GTT TCC CGA GGT-3′
NIK R7	5′-CAG CAT CAC ACT GAC ACT CAC C-3′	5′-TCC CTT CAC CCC ATC TCC T-3′
NIK R8	5′-TGT CCA GCT GTC CAC ATT GA-3′	5′-CAT CAC CCC AAA CTT TAT TGC TTA C-3′
Luciferase	5′-CGG AGG AGT TGT GTT TGT GG-3′	5′-ACT TTC CGC CCT TCT TGG-3′
AID	5′-CTG CAT GAA AAT TCA GTT CGT CTC-3′	5′-GCG TCT CGT AAG TCA TCA ACC TC-3′
MYB	5′-GCA TCA GAA GAT GAA GAC AAT GTT C-3′	5′-AGG ATG CAG GTT CCC AGG T-3′
IRF8	5′-CCT TGC GCT CCA AAC TCA TT-3′	5′-ACA GCT CTT CCC AGC CTC TTC-3′
PU.1	5′-GGC ACC TTC CAG TTC TCG T-3′	5′-GTA GGT CAT CTT CTT GCG GTT G-3′
BCL-xl	5′-ACC TGC CTG CCT TTG CCT AA-3′	5′-AAT AGG GAT GGG CTC AAC CAG TC-3′
CD83	5′-TGC TGG CTC TGG TTA TTT TCT ACT T-3′	5′-CTC GTT CCA TGC CAG CTT T-3′
CD86	5′-GAG AGG GAA GAG AGT GAA CAG ACC-3′	5′-AAA ACA CGC TGG GCT TCA TC-3′
XIAP	5′-ATA AAT CAC TTG AGG TTC TGG TTG C-3′	5′-GCG CCT TAG CTG CTC TTC A-3′

Primer List for 3′UTR Amplification (Gene Name: Sequence)

name	Forward	Reverse
CD79B 3′UTR	5′-ACT AGT CAT GAC CTG GGT GCA GGC TC-3′	5′-ACG CGT CTC AGG ACA CAC CGT TTA TTC C-3′
SYK 3′UTR	5′-ACT AGT GTA GCC ACC AAG GAG GGC AAA TAG-3′	5′-ACG CGT GTC CAG TGA CAC AAT GTA GCT C-3′
PLCG1 3′UTR	5′-ACT AGT TAC CCC AGC CTC GTT GGA GAG-3′	5′-ACG CGT GCA GCA GAA CTA GCA AAT GGC-3′
PKCbII 3′UTR 1	5′-ACT AGT GTA GAT GTG TAG ATC TCC GTC C-3′	5′-ACG CGT CAA CAG AAA CAA AAC CCC AAA GC-3′
PKCbII 3′UTR 2	5′-ACT AGT GAC ACC TCC AAC TTC GAC AAA G-3′	5′-ACG CGT CCT CCA AGA GAC AAT AGT TCA CAG-3′
IKKb 3′UTR	5′-ACG CGT GAC ATG GGG CAG CCC ATA GCA GG-3′	5′-AAG CTT GCC TGT AAC ATA AGC TCA CAG CC-3′
NIK 3′UTR	5′-GTA ACT AGT CCC TGC CCT CCA CCG CCG GCT-3′	5′-CGT AAG CTT GCC TGG AAA CAT TTG TCA TCA-3′
MYD88 3′UTR	5′-ACT AGT GAC TGT TCT GAG GCC CTG GGT GT-3′	5′-ACG CGT CTC AGG ATG CAA GAT ATA TTC CAG G-3′
PIK3CA 3′UTR	5′-ACG CGT GAA AGC TCA CTC TGG ATT CCA CAC-3′	5′-AAG CTT AAT TTT GAA ATG AAC TAG TTT AAG TGC-3′
PIK3CB 3′UTR	5′-ACT AGT CGA TCA GCC TTC GCT CCT AAT GT-3′	5′-ACG CGT CTC AAC CTT GTC TAT AGC TTA ATG CA-3′
PIK3CD 3′UTR	5′-ACT AGT GCC TCC CAA AGT GCT GGG ATT AC-3′	5′-ACG CGT CAG AGT GCT GGT CTC AAC CCA CAT-3′
PIK3CG 3′UTR	5′-ACT AGT CAA GTT AGT GTT CTA TGG TTT AAA TTA G-3′	5′-ACG CGT GAG ACT AAA CCC AGA TGA AAA TAG ATG-3′
RasGRP3 3′UTR	5′-GAG CTC CAG GCT GCG GAA ACT GAA GGC AA-3′	5′-ACG CGT CAC AGG AAG GCT GGC AAT ATA AAA CTT CCT A-3′

Primer Sequences for miR-31 Mutagenesis (Name: Sequence) (Underlines represent mutated sequence)

name	Forward	Reverse
miR-31-Mu1	5′-GAG GAC CGT TGA TGC TGG CAT AGC TGT T-3′	5′-GCA TCA ACG GTC CTC TCC AGT TCC AAG T-3′
miR-31-Mu2	5′-GAG GCA TCT TGC TGG CAT AGC TGT TGA-3′	5′-CCA GCA AGA TGC CTC CTC TCC AGT TCC-3′
miR-31-Mu3	5′-CAA GAT CGA GGC ATA GCT GTT GAA CTG-3′	5′-TAT GCC TCG ATC TTG CCT CCT CTC CAG-3′
miR-31-Mu4	5′-ATG CTC CGT AAG CTG TTG AAC TGG GAA C-3′	5′-CAG CTT ACG GAG CAT CTT GCC TCC TCT C-3′
miR-31-Mu5	5′-TGG CAT TCG TGT TGA ACT GGG AAC CTG-3′	5′-TCA ACA CGA ATG CCA GCA TCT TGC CTC-3′

PCR Primers for Amplification of miRNA-Encoding DNA Sequence and Subcloning into Lentiviral Vector (Name: Sequence)

name	Forward	Reverse
Pre-miR-200a	5′-AGA TCTA CCG GGC CCC TGT GAG CAT-3′	5′-TCT AGA AAA AAA GCG GGT CAC CTT TGA ACA TCG T-3′
Pre-miR-200b	5′-AGA TCT ACC AGC TCG GGC AGC CGT G-3′	5′-TCT AGA AAA AAA CGT GCA GGG CTC CGC CGT-3′
Pre-miR-200c	5′-AGA TCT ACC CTC GTC TTA CCC AGC AGT G-3′	5′-TCT AGA AAA AAA CCT CCA TCA TTA CCC GGC AGT-3′
Pre-miR-141	5′-AGA TCT ACG GCC GGC CCT GGG TCC A-3′	5′-TCT AGA AAA AAA GAA CCC ACC CGG GAG CCA TC-3′
Pre-miR-203	5′-AGA TCT AGT GTT GGG GAC TCG CGC G-3′	5′-TCT AGA AAA AAA TCG CTG TCG CCG CGC CC-3′
Pre-miR-205	5′-AGA TCT AAA GAT CCT CAG ACA ATC CAT GTG C-3′	5′-TCT AGA AAA AAA TGT CAG CTC CAT GCC TCC TG-3′
Pre-miR-214	5′-AGA TCT GGC CTG GCT GGA CAG AGT TG-3′	5′-TCT AGA AAA AAA GGC TGG GTT GTC ATG TGA CTG-3′
Pri-miR-200a	5′-AGA TCT GGT CCC GGC ACC ACC CCT-3′	5′-TCT AGA AAA AAA CCG CCC TCA CCC GTC TGC TG-3′
Pri-miR-200b	5′-AGA TCT GCT GTC CTC AGT GCC CCA GGA-3′	5′-TCT AGA AAA AAA GGT CCG GTG AGC GGG CTG TG-3′
Pri-miR-200c	5′-AGA TCT GTC CCC AGG GAC TCG GGT GGT-3′	5′-TCT AGA AAA AAA GAC GAG GGG CTT CCG GGG T-3′
Pri-miR-141	5′-AGA TCT CCT TGA GCT TGG GGT TGG CT-3′	5′-TCT AGA AAA AAA CCA GAG GGG TGA AGG TCA GAG GT-3′
Pri-miR-429	5′-AGA TCT CAG GGC AGC TGC AGG AGG T-3′	5′-TCT AGA AAA AAA GCC ATA GTG AGG GGG GCA TGA-3′
Pri-miR-203	5′-AGA TCT GTC CCC GGG ATC CGC AGG CGA-3′	5′-TCT AGA AAA AAA CCT GGA GCG CGG AGC CGG T-3′
Pri-miR-205	5′-AGA TCT GGA TGC CAA AGG CCT TTG GCC A-3′	5′-TCT AGA AAA AAA CTC TTG CTG CTG CTG CTG CT-3′
Pri-miR-31	5′-AGA TCT GTG CGC TTT CAA TTA ATG AGT GT-3′	5′-TCT AGA AAA AAA GGT GAG AAA GGC CAT GTC AT-3′
Pri-miR-135b	5′-AGA TCT GGG TCG GGG GTG CTG AGA A-3′	5′-TCT AGA AAA AAA GAT GCC CCA CCC CCA AGC A-3′

Insert Sequences for Lentiviral Vector (Name: Sequence) (Underlines represent antisense of shRNA target sequences)

name	Sequence
LV-miR-31-top	5′-GAT CCC CGG AGA GGA GGC AAG ATG CTG GCA TAG CTG TTG AAC TGG GAA CCT GCT ATG CCA ACA TAT TGC CAT CTT TCC TTT TTG GAA AT-3′
LV-miR-31-bottom	5′-CTA GAT TTC CAA AAA GGA AAG ATG GCA ATA TGT TGG CAT AGC AGG TTC CCA GTT CAA CAG CTA TGC CAG CAT CTT GCC TCC TCT CCG GG-3′
LV-shCtrl-top	5′-GAT CCC CCA TCG ACT GAA ATC CCT GGT AAT CCG TTG TTA ACA ACG GAT TAC CAG GGA TTT CAG TCG ATG TTT TTG GAA AT-3′
LV-shCtrl-bottom	5′-CTA GAT TTC CAA AAA CAT CGA CTG AAA TCC CTG GTA ATC CGT TGT TAA CAA CGG ATT ACC AGG GAT TTC AGT CGA TGG GG-3′
LV-shAgo2#1-top	5′-GAT CCC CGC AGG ATA AGG ATG TGT TAA CGT GTG CTG TCC GTT AAT ACA TCT TTG TCC TGC TTT TTG GAA AT-3′
LV-shAgo2#1-bottom	5′-CTA GAT TTC CAA AAA GCA GGA CAA AGA TGT ATT AAC GGA CAG CAC ACG TTA ACA CAT CCT TAT CCT GCG GG-3′
LV-shAgo2#2-top	5′-GAT CCC CGC ATG GAA GTC TAT CTG GAA CGT GTG CTG TCC GTT TCA GAT GGA CTT CCG TGC TTT TTG GAA AT-3′
LV-shAgo2#2-bottom	5′-CTA GAT TTC CAA AAA GCA CGG AAG TCC ATC TGA AAC GGA CAG CAC ACG TTC CAG ATA GAC TTC CAT GCG GG-3′
LV-shDicer#1-top	5′-GAT CCC CGC TTG AAG TCT TAC GCA GAT AAC GTG TGC TGT CCG TTA TTT GCG TAA GAT TTC GAG CTT TTT GGA AAT-3′
LV-shDicer#1-bottom	5′-CTA GAT TTC CAA AAA GCT CGA AAT CTT ACG CAA ATA ACG GAC AGC ACA CGT TAT CTG CGT AAG ACT TCA AGC GGG-3′
LV-shDicer#2-top	5′-GAT CCC CGC CGA GGA AGT CAG CTG AAT TAC GTG TGC TGT CCG TAA TTT AGC TGA TTT CCT TGG CTT TTT GGA AAT-3′
LV-shDicer#2-bottom	5′-CTA GAT TTC CAA AAA GCC AAG GAA ATC AGC TAA ATT ACG GAC AGC ACA CGT AAT TCA GCT GAC TTC CTC GGC GGG-3′
LV-shTRBP-top	5′-GAT CCC CGC TGT CTA GTG TAG AGT AAA CGT GTG CTG TCC GTT TGC TCT ATA CTA GGC AGC TTT TTG GAA AT-3′
LV-shTRBP-bottom	5′-CTA GAT TTC CAA AAA GCT GCC TAG TAT AGA GCA AAC GGA CAG CAC ACG TTT ACT CTA CAC TAG ACA GCG GG-3′
LV-shCD79B-top	5′-GAT CCC CAC AGT CAC CTG TGA GGA CGT AAC GTG TGC TGT CCG TTA TGT CCT CAT AGG TGG CTG TTT TTT GGA AAT-3′
LV-shCD79B-bottom	5′-CTA GAT TTC CAA AAA ACA GCC ACC TAT GAG GAC ATA ACG GAC AGC ACA CGT TAC GTC CTC ACA GGT GAC TGT GGG-3′
LV-shSYK-top	5′-GAT CCC CGG GTA TGA GTG GTG GGC TTT AAC GTG TGC TGT CCG TTA AAG CCC ATC ACT CAT GCC CTT TTT GGA AAT-3′
LV-shSYK-bottom	5′-CTA GAT TTC CAA AAA GGG CAT GAG TGA TGG GCT TTA ACG GAC AGC ACA CGT TAA AGC CCA CCA CTC ATA CCC GGG-3′
LV-shPLCG1-top	5′-GAT CCC CGC TAT TGA CGT TCG TGA GAT TAC GTG TGC TGT CCG TAA TTT CAC GAA TGT CAA TGG CTT TTT GGA AAT-3′
LV-shPLCG1-bottom	5′-CTA GAT TTC CAA AAA GCC ATT GAC ATT CGT GAA ATT ACG GAC AGC ACA CGT AAT CTC ACG AAC GTC AAT AGC GGG-3′
LV-shPKCb-top	5′-GAT CCC CGC TGG AAG AAT TGG ACA GAG AAC GTG TGC TGT CCG TTC TTT GTC CGA TTC TTT CAG CTT TTT GGA AAT-3′
LV-shPKCb-bottom	5′-CTA GAT TTC CAA AAA GCT GAA AGA ATC GGA CAA AGA ACG GAC AGC ACA CGT TCT CTG TCC AAT TCT TCC AGC GGG-3′
LV-shIKKb-top	5′-GAT CCC CGT TGG TTC GTA TCT TGA GCA TAC GTG TGC TGT CCG TAT GTT CAA GAT ATG AAC CAG CTT TTT GGA AAT-3′
LV-shIKKb-bottom	5′-CTA GAT TTC CAA AAA GCT GGT TCA TAT CTT GAA CAT ACG GAC AGC ACA CGT ATG CTC AAG ATA CGA ACC AAC GGG-3′
LV-shNIK-top	5′-GAT CCC CGC CGG TCT GAG GGT CTT GAA CGT GTG CTG TCC GTT CAA GAC TCT CGG ACT GGC TTT TTG GAA AT-3′
LV-shNIK-bottom	5′-CTA GAT TTC CAA AAA GCC AGT CCG AGA GTC TTG AAC GGA CAG CAC ACG TTC AAG ACC CTC AGA CCG GCG GG-3′
LV-shRasGRP1-top	5′-GAT CCC CGC TGT CTC TAG AGC AGG ACT AAC GTG TGC TGT CCG TTA GTC TTG CTT TAG AGG CAG CTT TTT GGA AAT-3′
LV-shRasGRP1-bottom	5′-CTA GAT TTC CAA AAA GCT GCC TCT AAA GCA AGA CTA ACG GAC AGC ACA CGT TAG TCC TGC TCT AGA GAC AGC GGG-3′
LV-shRasGRP3-top	5′-GAT CCC CGC TGT AAT GAG TTT CGG TTA AAC GTG TGC TGT CCG TTT AAT CGA AAT TCA TTG CAG CTT TTT GGA AAT-3′
LV-shRasGRP3-bottom	5′-CTA GAT TTC CAA AAA GCT GCA ATG AAT TTC GAT TAA ACG GAC AGC ACA CGT TTA ACC GAA ACT CAT TAC AGC GGG-3′
